# Increased Body Mass Index and Risk of Left Atrial Thrombus in Nonvalvular Atrial Fibrillation Patients—Data from the Left Atrial Thrombus on Transesophageal Echocardiography (LATTEE) Registry

**DOI:** 10.3390/nu14173652

**Published:** 2022-09-04

**Authors:** Beata Uziębło-Życzkowska, Agnieszka Kapłon-Cieślicka, Marek Kiliszek, Monika Gawałko, Monika Budnik, Katarzyna Starzyk, Beata Wożakowska-Kapłon, Ludmiła Daniłowicz-Szymanowicz, Damian Kaufmann, Maciej Wójcik, Robert Błaszczyk, Jarosław Hiczkiewicz, Katarzyna Łojewska, Katarzyna Mizia-Stec, Maciej T. Wybraniec, Katarzyna Kosmalska, Marcin Fijałkowski, Anna Szymańska, Aleksandra Gos, Maciej Haberka, Michał Kucio, Błażej Michalski, Karolina Kupczyńska, Anna Tomaszuk-Kazberuk, Katarzyna Wilk-Śledziewska, Renata Wachnicka-Truty, Marek Koziński, Paweł Burchardt, Paweł Krzesiński

**Affiliations:** 1 Department of Cardiology and Internal Diseases, Military Institute of Medicine, 01-755 Warsaw, Poland; 2 “Club 30”, Polish Cardiac Society, 40-001 Katowice, Poland; 3 1st Chair and Department of Cardiology, Medical University of Warsaw, 02-091 Warsaw, Poland; 4 Institute of Pharmacology, West German Heart and Vascular Centre, University Duisburg-Essen, 4514 Essen, Germany; 5 Department of Cardiology, Maastricht University Medical Centre and Cardiovascular Research Institute Maastricht, 6229 ER Maastricht, The Netherlands; 6 Collegium Medicum, Jan Kochanowski University, 25-317 Kielce, Poland; 7 Department of Cardiology and Electrotherapy, Medical University of Gdansk, 80-210 Gdansk, Poland; 8 Department of Cardiology, Medical University of Lublin, 20-059 Lublin, Poland; 9 Collegium Medicum, University of Zielona Góra, 65-417 Zielona Gora, Poland; 10 Clinical Department of Cardiology, Nowa Sól Multidisciplinary Hospital, 67-100 Nowa Sol, Poland; 11 1st Department of Cardiology, School of Medicine in Katowice, Medical University of Silesia, 40-635 Katowice, Poland; 12 Members of the European Reference Network on Heart Diseases—Ern Guard-Heart, 1105 AZ Amsterdam, The Netherlands; 13 Department of Cardiology, St. Vincent Hospital, 81-348 Gdynia, Poland; 14 1st Department of Cardiology, Medical University of Gdansk, 80-210 Gdansk, Poland; 15 Department of Heart Diseases, Postgraduate Medical School, 00-002 Warsaw, Poland; 16 Department of Cardiology, School of Health Sciences, Medical University of Silesia, 40-055 Katowice, Poland; 17 Department of Cardiology, Medical University of Lodz, 90-419 Lodz, Poland; 18 Department of Cardiology, Medical University of Bialystok, 15-089 Bialystok, Poland; 19 Department of Cardiology and Internal Medicine, Medical University of Gdansk, 80-210 Gdansk, Poland; 20 Department of Hypertension, Angiology, and Internal Medicine, Poznan University of Medical Sciences, 61-701 Poznan, Poland

**Keywords:** atrial fibrillation, atrial flutter, body mass index, left atrial thrombus

## Abstract

An increased body mass index (BMI) is associated with a higher incidence of atrial fibrillation (AF) and a higher risk of thromboembolic complications in AF patients. The aim of this study was to investigate the effect of BMI on the risk of left atrial thrombi (LATs) in patients with nonvalvular AF/atrial flutter (AFl) (NV AF/AFl). Patients diagnosed with NVAF/AFl (between November 2018 and May 2020) were selected from the multicenter, prospective, observational Left Atrial Thrombus on Transesophageal Echocardiography (LATTEE) registry that included AF/AFl patients referred for cardioversion or ablation followed by transesophageal echocardiography. A total of 2816 AF/AFl patients (63.6% males; mean age 65.8 years; mean BMI 29.8 kg/m^2^) were included in the study. Two hundred and twenty-two of them (7.9%) had LATs. Compared with normal-weight patients, those with BMIs ≥ 25 kg/m^2^ more frequently presented clinical factors potentially provoking LATs, such as non-paroxysmal AF/AFl (*p* = 0.04), hypertension (*p* < 0.001), and diabetes (*p* < 0.001); had higher CHA_2_DS_2_ scores (*p* < 0.001); and had larger LA dimensions (LA diameter and LA area) (*p* < 0.001 for both parameters). On the other hand, they showed some features negatively related to thromboembolic risk; for example, they were younger (*p* < 0.001) and were more often male (*p* = 0.002). In addition, patients with abnormal BMIs were more likely to be smokers (*p* = 0.006) and to be treated with oral anticoagulants (*p* = 0.005). Despite these differences in the prevalence of thromboembolic risk factors, the incidence of LATs was not increased in patients with abnormal body weight (overweight and obese compared to normal-weight patients) in this large real-life cohort of AF/AFl patients. This is probably due to the balanced composition regarding the prevalence of positive and negative thromboembolic risk factors.

## 1. Introduction

As known from the Framingham cohort population, the incidence of atrial fibrillation (AF) increases by 4% with every one-unit increase in body mass index (BMI) [1]. In AF patients, abnormal body weight has been shown to be associated with known thromboembolic risk factors (CHA_2_DS_2_-VASc score), such as hypertension, diabetes, and heart failure [2]. Some researchers have linked obesity to hypofibrinolysis, inflammation, and prothrombotic conditions, which may additionally indicate an association with thromboembolism [3]. However, the relationship between obesity and thromboembolism in AF as an independent risk factor is still contentious [4,5,6]. No body mass index is included in the well-established stroke risk scores [4]. Despite the potential influence of obesity on the pharmacokinetics of anticoagulants, there is also no evidence that their doses should be modified if applied according to guidelines [4,7].

Cardiogenic strokes in patients with AF are most commonly associated with thrombi in the left atria (LA), particularly in the LA appendage (LAA). One of the recent studies shows that abnormal weight increases the risk of thrombus formation in the LAA [8], but the data are inconclusive [9].

Therefore, our study aimed to assess whether an increased BMI is associated with an increased risk of left atrial thrombus (LAT) formation in a large population of nonvalvular atrial fibrillation/atrial flutter (NV AF/AFl) patients.

## 2. Methods

### 2.1. Patients

**Figure 1:**
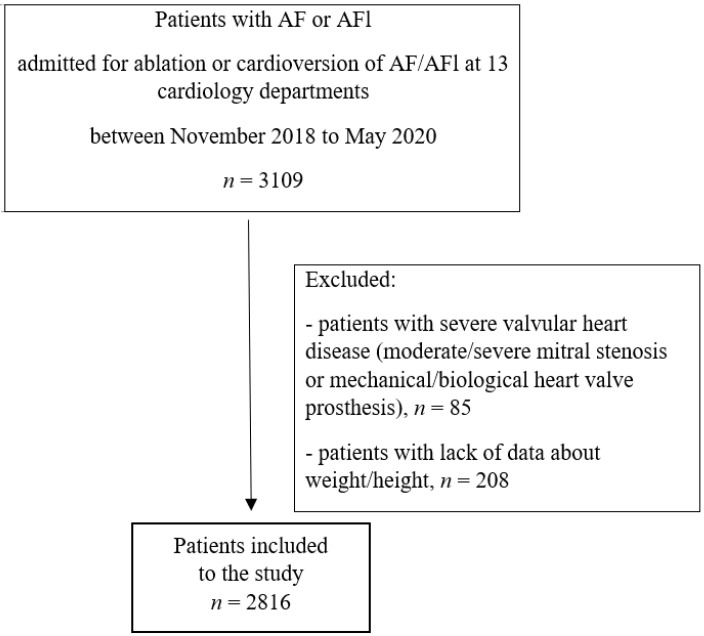
The flowchart of the study. Abbreviations: AF, atrial fibrillation; AFl, atrial flutter.

In this prospective, observational, and multicenter study, we analyzed numerous data collected from consecutive AF and AFl patients enrolled at 13 cardiology centers (the Left Atrial Thrombus on TransEsophageal Echocardiography (LATTEE) registry; NCT03591627). All patients admitted for electrical cardioversion and/or catheter ablation who underwent transesophageal echocardiography (TEE) were included in our study. The LATTEE study methods have been described in detail [10,11]. In summary, all patients admitted for AF/AFl ablation were included in the study. Regarding non-emergency electrical cardioversion due to AF/AFl, four cardiology centers performed TEE on all patients, and nine centers performed TEE only on the patients suspected of not having received anticoagulant treatment in the previous three weeks. Finally, out of 3109 patients, 2816 were included in this analysis (Figure 1). The study was conducted according to clinical practice guidelines and the Declaration of Helsinki and was approved by the ethics committee (AKBE/113/2018). Data were entered into the registry database anonymously. The ethics committee waived the requirement to obtain informed consent from the patients.

### 2.2. Data Collection

Demographic, clinical, laboratory, and echocardiographic data were collected prospectively for all the enrolled patients. BMI was calculated as weight in kilograms divided by the square of height in meters, measured at the time of admission. The presence of AF/AFl was confirmed by electrocardiography. BMI was evaluated as a categorical variable (normal weight is defined as <25 kg/m^2^; overweight as 25.0–29.99 kg/m^2^; and obesity as ≥ 30 kg/m^2^). The overweight and obese groups were considered to have abnormal weight. The diagnostic criteria for heart failure (HF) in AF and AFl patients were adopted as recommended in the ESC guidelines [4,12]. The estimated glomerular filtration rate (eGFR) was calculated using the CKD-EPI formula to avoid overestimating the eGFR in obese subjects. The CHA_2_DS_2_ and CHA_2_DS_2_-VASc scores were calculated according to the current guidelines [4].

Transesophageal echocardiography was performed by certified echocardiographers before direct current cardioversion or catheter ablation. The presence of thrombi in both the LA and LAA was evaluated. An LA thrombus was identified as a circular or irregular echodense mass in the LA or LAA that was not part of the endocardium or pectinate muscles [13]. The LA appendage-emptying velocity was measured 1 cm below the orifice of the appendage.

Data from the transthoracic echocardiography were collected and included the left atrial end-diastolic diameter (Lad), obtained from the parasternal longitudinal axis view; left atrial area (LAa), measured from the apical four-chamber view; and left ventricular ejection fraction (LVEF), calculated using the biplane Simpson formula. All the echocardiographic measurements were conducted according to the current guidelines [14]. 

### 2.3. Statistical Analysis

Statistical analyses were conducted with IBM SPSS Statistics 25 (SPSS Inc., Chicago, IL, USA). The data are presented as the medians and interquartile ranges or as the numbers of patients and percentages, where appropriate. The chi-squared test was used to test the relationship between nominal variables and to check whether the compared groups were equal. The Mann–Whitney U test was used to evaluate the statistical significance of differences between two independent groups. The Kruskal–Wallis test was used to assess the statistical significance of differences between more than two groups. If there were such differences, an appropriate post hoc test was used. The effect size of the risk factor for thrombus occurrence was measured using the eta-squared ratio. A *p*-value < 0.05 was considered statistically significant.

## 3. Results

### 3.1. General Study Population

A total of 2816 AF or AFl patients were included in the study. Only 474 (16.8%) subjects had normal weight. As many as 2342 (83.2%) patients had BMIs above normal (defined as > 24.99 kg/m^2^), the vast majority of which were men (64.9%). Patients with a BMI > 40 kg/m^2^ accounted for 2.8% and those with a BMI < 25 kg/m^2^ represented only 0.003%.

Detailed characteristics of the patients by BMI category are presented in Table 1 and Table 2. As expected, the prevalence of hypertension (1870 (79.9%) vs. 278 (58.6%); *p* < 0.001) and diabetes mellitus (646 (27.6%) vs. 66 (13.9%); *p* < 0.001) was higher in the patients with abnormal BMIs (defined as BMI > 24.99 kg/m^2^). Larger LA dimensions (LAd and LAa) were also characteristic of this patient group (*p* < 0.001 for both parameters). However, the differences in these parameters were most significant for the obese group (*p* < 0.001 for both parameters vs. overweight group and vs. normal-weight group). Smoking was also significantly more common in the patients with abnormal body weight (*p* = 0.006). Concerning HF with a preserved ejection fraction (HFpEF), differences were mainly observed for obese patients compared to the other analyzed groups (241 (19.1%) for obese vs. 153 (14.2%) for overweight vs. 63 (13.4%) for normal weight; *p* = 0.001). The durations of AF/AFl were comparable between all the groups of patients (*p* = 0.31). Non-paroxysmal AF/AFl was significantly more frequent (*p* < 0.001 vs. both other groups) in obese NVAF/AFl patients (with BMI > 29.99 kg/m^2^). Overweight and obese patients had significantly higher CHADS_2_ (*p* < 0.001), but the CHA_2_DS_2_-VASc scores were significantly different only for obese patients versus the rest of the study group (*p* = 0.02 for the overweight group and *p* = 0.01 for the normal-weight group). The hemoglobin level was significantly higher in the abnormal-weight patients (*p* < 0.001), especially in the obese group vs. other groups of patients (*p* = 0.01 vs. overweight group and vs. normal group). 

### 3.2. BMI and the Risk of Left Atrial Thrombus

LATs were detected in 222 (7.9%) study patients (6.9% in patients on chronic OAC). Despite the above differences, overweight and obese patients had no difference in LAT incidence compared to normal-weight NVAF/AFl patients (overweight: 8.2%; obese: 7.3% vs. normal weight: 8.9%; *p* = 0.5) (Table 3). Likewise, no difference was observed when comparing the groups of patients with abnormal vs. normal weight (7.7% vs. 8.9%, respectively; *p* = 0.4) (Table 3). Importantly, the obese and overweight groups were more likely to be undergoing oral anticoagulant (OAC) treatment in general (*p* = 0.005). Concerning anticoagulants, apixaban was significantly less frequently chosen in the abnormal-weight group (*p* = 0.02), whereas rivaroxaban was more frequently used (*p* = 0.01).

## 4. Discussion

In our real-world population of AF/AFl patients referred for TEE prior to cardioversion or ablation, the incidence of LA thrombus was 7.9%. We realized that it is higher than the one reported in the largely anticoagulated patient population [15]. However, the aforementioned large meta-analysis of randomized controlled trials performed by Lurie et al. [16] included only AF/AFl patients who were on oral anticoagulation with NOACs or VKAs in addition to a confirmed minimum of 3 weeks of continuous VKA/NOAC treatment. Our study included non-selected, consecutively hospitalized AF/AFl patients with no exclusion criteria. This is the uniqueness of our real-world study, which means that the results cannot be compared with large meta-analyses of randomized trials. Moreover, in our study, only 173 (6.9%) cases of LAT were found in patients on chronic OAC. These findings are similar to those reported in a large recent meta-analysis, which included 85 studies and 56,660 patients with AF (16). In the mentioned study, the prevalence of LA thrombus in 9772 anticoagulated patients undergoing TEE before electrical cardioversion or catheter ablation was 6.7% (95% confidence interval [CI] 4.3–9.7%). 

The main finding of our study is that an increased BMI is not associated with an increased risk of LAT in patients with nonvalvular AF undergoing ablation or cardioversion, most of whom were receiving anticoagulation. The absence of this relationship was found both when comparing the different study groups among themselves (normal weight vs. overweight vs. obese) and after dividing the study group into patients with normal vs. abnormal (overweight and obese together) body weight. Our results are comparable to those of Cohoon et al. [9], except that they examined a naïve NVAF anticoagulation cohort referred for TEE. Here, the abnormal-weight group received OAC treatment more frequently than the normal-weight patients. We also showed that there were differences in the use of individual anticoagulants between the normal weight group and those with abnormal weight. Apixaban was significantly less frequently chosen in the abnormal-weight group (*p* = 0.02), and rivaroxaban was used more frequently (*p* = 0.01). It is difficult to say conclusively whether this difference could have biased our results, as the majority in both subgroups were receiving the treatment (89% vs. 84%). In addition, in a summary of studies involving anticoagulants in the AF patient population, the guideline authors indicate that no differences were apparent with regard to the incidence of thromboembolic endpoints in obese patients treated with NACs compared to patients without obesity [7]. In the case of patients with a BMI ≥ 40 kg/m^2^, the available research data are less reliable. However, in our study, patients with BMI > 40 kg/m^2^ comprised only 2.8%, which seems unlikely to disturb the results. 

In contrast, Tang et al. [8] demonstrated that an increased BMI may be associated with an increased risk of thrombus formation in the LA appendage, with a BMI of 27 kg/m^2^ as the cut-off point. The incidence of LAT/LAAT was 10.6% in patients with BMIs > 27.0 kg/m^2^ and 3.0% in patients with BMIs < 27.0 kg/m^2^ (*p* = 0.001). This study mostly included patients not treated with OACs. Fewer than 3% of the ablation patients took warfarin, and fewer than 20% took aspirin. The study groups in both mentioned studies were significantly smaller than ours; moreover, the study by Tang et al. [8] was published in 2009, when the prevalence of NOAC use was significantly lower. 

As with most studies, our results showed that an increased BMI is associated with a higher prevalence of hypertension, diabetes mellitus, non-paroxysmal AF, LA enlargement, and higher CHADS_2_ scores (and CHA_2_DS_2_-VASC scores for obese patients) [17,18,19,20]. These conditions increase the risk of LAT formation, some of which are part of the CHA_2_DS_2_-VASC score [21,22]. Non-paroxysmal AF, however, is not a part of the CHA_2_DS_2_-VASC score and is a significant risk factor for LAT formation. Kapłon-Cieślicka et al. [23] proposed including this parameter in existing thromboembolic risk scales (as a new CHA_2_DS_2_VASc-RAF score, with R for renal function and AF for non-paroxysmal AF). Another factor associated with an increased thromboembolic risk more frequently identified in our AF/AFl patients with increased BMIs was smoking. The results of previous studies indicate that active smokers have reduced LAA velocities and are also more likely to have spontaneous contrast in the LAA [24]. Furthermore, in a large cohort of young patients (57,053 people: 27,178 men; 29,876 women; aged 50 to 64 years), Albertsen et al. [25] showed that the risk ratios (HRs) (95% CIs) for thromboembolism or death were 3.13 (1.72–6.37) and 2.73 (2.02–3.70), respectively, for women and men who were current heavy smokers (>25 g/d). Furthermore, these relationships remained after adjusting for established thromboembolic risk factors (HRs of 3.64 (1.88–7.07) and 2.17 (1.59–2.95) among women and men, respectively).

Despite the association of the abovementioned thromboembolic risk factors with increased BMIs, our cohort’s AF/AFl patients with abnormal and normal body mass presented a similar incidence of LAT. This might be partly explained by the lower proportion of women and the younger ages of the overweight patients compared to those of the normal-weight patients. Older age and female sex are recognized thromboembolic risk factors and are strongly represented in scales such as the CHA_2_DS_2_-VASc.

Finally, it is worth dedicating a few sentences to the relationship postulated in the literature between LAT frequency and chronic kidney disease. Chronic kidney disease and reduced eGFR are not included in existing thromboembolic risk scales, but many studies postulated that this factor significantly increases the incidence of LAT formation [23,26,27]. In our study, obese patients had slightly worse renal function compared to normal-weight and overweight patients, but this difference was not statistically significant. Despite the slightly worse renal function in obese patients, the incidence of LAT did not appear to be higher in this group.

## 5. Conclusions

The most important finding of our study is that there was no association between an increased BMI and a higher incidence of LAT in a large real-life cohort of AF/AFl patients. This lack of correlation was found even though the patients with abnormal body weight, although younger and more frequently male, presented a higher prevalence of multiple well-established thromboembolic risk factors than the normal-weight patients. 

### Limitations

Our study has some limitations. Firstly, although we included a relatively large population of patients with AF/AFl, these were only patients admitted for ablation or cardioversion procedures. Therefore, the results cannot be extrapolated to the whole population of patients with NVAF/AFl. Secondly, we did not follow up with patients, so we did not assess the incidence of thromboembolic events per se but only assessed the presence of LAT, taking it as a surrogate for high thromboembolic risk. Due to the registered nature of the study, it was unavoidable that the subgroups were not perfectly balanced by age and sex and that certain parameters were missing and, therefore, could not be included in the analysis. Finally, the limitations of using BMI alone as an indicator of obesity-type disorders should be mentioned. We are aware that it is not an ideal indicator of metabolic disorders. The study did not assess obesity type, nor did it measure waist-to-hip ratio.

## Figures and Tables

**Table 1:** Characteristics of the study group according to body mass index category.

Variable		BMI (kg/m^2^)			*p*-Value
		<25 N = 474; 16.8% Normal Weight	25.0–29.9 N = 1077; 38.2% Overweight	>30 N = 1265; 44.9% Obese	
The reason for admission					
Catheter ablation		219 (47)	517 (48.8)	580 (46.4)	0.51
Cardioversion		247 (53)	542 (51.2)	669 (53.6)	0.51
Type of AF/AFl					
AF/AFl non-paroxysmal		261 (55.2)	605 (56.4)	805 (63.8)	<0.001
Time from AF/AFl diagnosis (years)		2 (1–5)	2 (1–5)	2 (1–5)	0.31
Demographic data					
Age (years)		68 (60–76)	67 (60–73)	66 (59–72)	<0.001
Sex	Female	202 (42.6)	363 (33.7)	460 (36.4)	0.004
	Male	272 (57.4)	714 (66.3)	805 (63.6)	
Concomitant diseases					
Heart failure		193 (41)	447 (41.5)	564 (44.7)	0.2
HFrEF		75 (15.9)	171 (15.9)	187 (14.8)	0.73
HFmrEF		56 (11.9)	128 (11.9)	140 (11.1)	0.81
HFpEF		63 (13.4)	153 (14.2)	241 (19.1)	0.001
Hypertension		278 (58.6)	809 (75.1)	1061 (83.9)	<0.001
Diabetes mellitus		66 (13.9)	224 (20.8)	422 (33.4)	<0.001
Previous TIA		14 (3)	31 (2.9)	37 (2.9)	0.99
Previous stroke		35 (7.4)	85 (7.9)	89 (7)	0.74
Coronary artery disease		130 (27.4)	328 (30.5)	379 (30)	0.47
Chronic kidney disease		83 (17.5)	154 (14.3)	220 (17.4)	0.09
Smoking		135 (28.7)	346 (32.9)	462 (37.3)	0.002
Malignant tumor		15 (3.2)	37 (3.4)	40 (3.2)	0.93
COPD		32 (6.8)	50 (4.6)	67 (5.3)	0.23
CHA_2_DS_2_-VASc score (points)		3 (2–4)	3 (2–4)	3 (2–5)	0.002
CHADS_2_ score (points)		1 (1–3)	2 (1–3)	2 (1–3)	<0.001
Laboratory and echocardiography data					
Hemoglobin (g/dL)		13.7 (12.8–14.7)	14.1 (13–15.1)	14.3 (13.2–15.3)	<0.001
eGFR (mL/min/1.73 m^2^)		59.8 (46.5–80.9)	59.1 (48.7–78.5)	57.1 (45.6–77.2)	0.07
LVEF (%)		55 (42–60)	55 (45–60)	55 (45–60)	0.85
LAd (cm)		43 (39–47)	45 (41–48.8)	46 (43–50)	<0.001
LA area (cm^2^)		24.9 (21.2–29)	25.4 (21.5–30)	27 (23.4–31)	<0.001
LAA emptying velocity (cm/s)		37 (25–53.3)	40 (26–57)	38 (27–50)	0.74
Oral anticoagulation therapy					
OAC therapy		401 (84.6)	954 (88.7)	1132 (89.5)	0.02
VKA		54 (13.3)	161 (16.6)	182 (15.8)	0.3
warfarin		21 (4.4)	66 (6.1)	86 (6.8)	0.19
acenocoumarol		33 (7)	95 (8.8)	96 (7.6)	0.37
NOAC		352 (86.7)	807 (83.4)	972 (84.2)	0.3
rivaroxaban		142 (30)	397 (36.9)	449 (35.5)	0.03
dabigatran		132 (27.8)	289 (26.9)	352 (27.8)	0.86
apixaban		78 (16.5)	121 (11.3)	171 (13.5)	0.02

Data are presented as medians (interquartile ranges (IQRs), equal to the difference between the upper and lower quartiles) and n (%). Abbreviations: AF, atrial fibrillation; AFl, atrial flutter; BMI, body mass index; COPD, chronic obstructive pulmonary disease; eGFR, estimated glomerular filtration rate; HFmrEF, heart failure with mildly reduced ejection fraction; HFpEF, heart failure with preserved ejection fraction; HFrEF, heart failure with reduced ejection fraction; LA, left atrial; LAd, left atrial diameter; LAA, left atrial appendage; LVEF, left ventricular ejection fraction; NOAC, non-vitamin K-antagonist oral anticoagulants; VKA, vitamin K anticoagulants.

**Table 2:** Characteristics of the study group according to body mass index category (normal vs. abnormal weight (overweight + obese)).

Variable		BMI (kg/m^2^)		*p*-Value
		<25 N = 474; 16.8% Normal Weight	>24.99 N = 2342; 83.2% Overweight/Obese	
The reason for admission				
Catheter ablation		219 (47)	1097 (47.5)	0.83
Cardioversion		247 (53)	1211 (52.5)	0.83
Type of AF/AFl				
AF/AFl non-paroxysmal		261 (55.2)	1410 (60.4)	0.04
Time from AF/AFl diagnosis (years)		2 (1–5)	2 (1–5)	0.57
Demographic data				
Age (years)		68 (60–76)	67 (59–73)	<0.001
Sex	Female	202 (42.6)	823 (35.1)	0.002
	Male	272 (57.4)	1519 (64.9)	
Concomitant diseases				
Heart failure		193 (41)	1011 (43.3)	0.39
HFrEF		75 (15.9)	358 (15.3)	0.73
HFmrEF		56 (11.9)	268 (11.5)	0.81
HFpEF		63 (13.4)	394 (16.9)	0.07
Hypertension		278 (58.6)	1870 (79.9)	<0.001
Diabetes mellitus		66 (13.9)	646 (27.6)	<0.001
Previous TIA		14 (3)	48 (2.9)	0.88
Previous stroke		35 (7.4)	174 (7.4)	1
Coronary artery disease		130 (27.4)	707 (30.2)	0.25
Chronic kidney disease		83 (17.5)	374 (16)	0.41
Smoking		135 (28.7)	808 (35.3)	0.006
Malignant tumor		15 (3.2)	77 (3.3)	1
COPD		32 (6.8)	117 (5)	0.14
CHA_2_DS_2_-VASc score (points)		3 (2–4)	3 (2–4)	0.1
CHADS_2_ score (points)		1 (1–3)	2 (1–3)	<0.001
Laboratory and echocardiography data				
Hemoglobin (g/dL)		13.7 (12.8–14.7)	14.2 (13.1–15.2)	<0.001
eGFR (mL/min/1.73 m)		59.8 (46.5–80.9)	58 (46.9–78.2)	0.35
LVEF (%)		55 (42–60)	55 (45–60)	0.65
LAd (cm)		43 (39–47)	46 (42–50)	<0.001
LA area (cm^2^)		24.9 (21.2–29)	26.5 (22.5–30.3)	<0.001
LAA emptying velocity (cm/s)		37 (25–53.2)	39 (26–54)	0.79
Oral anticoagulation therapy				
OAC therapy		401 (84.6)	2086 (89.1)	0.005
VKA		54 (13.3)	343 (16.2)	0.16
warfarin		21 (4.4)	152 (6.5)	0.09
acenocoumarol		33 (7)	191 (8.2)	0.4
NOAC		352 (86.7)	1779 (83.8)	0.16
rivaroxaban		142 (30)	846 (36.2)	0.01
dabigatran		132 (27.8)	641 (27.4)	0.87
apixaban		78 (16.5)	292 (12.5)	0.06

Data are presented as medians (interquartile ranges (IQRs), equal to the differences between the upper and lower quartiles) and n (%). Abbreviations: as in Table 1.

**Table 3:** Prevalence of left atrial thrombus in relation to BMI.

Variable	LAT(−)	LAT(+)	*p*-Value
**BMI (kg/m^2^)**			
<25	432 (91.1)	42 (8.9)	0.5
25.0–29.9	989 (91.8)	88 (8.2)	
>30	1173 (92.7)	92 (7.3)	
**BMI (kg/m^2^)**			
<25	432 (91.1)	42 (8.9)	0.4
>24.99	2162 (92.3)	180 (7.7)	

Abbreviations as in Table 1. Data are presented as n (%).

## Data Availability

The data are available upon reasonable request to the corresponding author.
